# Effect of cyp1a1, cyp1b1 and cyp2c gene polymorphisms on doxorubicin and paclitaxel

**DOI:** 10.6026/9732063002001244

**Published:** 2024-10-31

**Authors:** Rashmi A. Gudur, Suresh J. Bhosale, Anand K. Gudur, Kailas D. Datkhile

**Affiliations:** 1 Department of Oncology, Krishna Vishwa Vidyapeeth (Deemed to be University), Karad, Satara - 415 539, Maharashtra, India; 2 Department of Molecular Biology and Genetics, Krishna Vishwa Vidyapeeth (Deemed to be University), Karad, Satara - 415 539, Maharashtra, India

**Keywords:** Breast cancer, gene polymorphisms, CYP1A1, CYP1B1, CYP2C, chemotherapy, toxicity

## Abstract

The genes encoding metabolizing cytochrome P450 enzyme are studied for their importance in cancer susceptibility. Therefore, it is of interest to identify the correlation of CYP1A, CYP1B and CYP2C gene polymorphisms on drug response (DG-RS) and toxicity reactions in Indian population. Hence, 200 breast cancer patients received doxorubicin (DXR) and paclitaxel (PCX) chemotherapy. Further, chemotherapy induced hematological (HEM) and none (N)-HEM toxicity reactions were recorded. We found that, the Univariate Logistic Regression analysis showed negative association of CYP1B1 (4326 C>G) gene polymorphisms with microsites (OR=0.14, 95% CI: 0.03-0.54; p=0.004) in breast cancer patients treated with Doxorubicin. Thus, protective effect of CYP1B1-polymorphisms with doxorubicin and paclitaxel based chemotherapy induced N-HEM toxicity and CYP2C9- polymorphisms with paclitaxel induced body ache and CYP1A1-polymorphisms with peripheral neuropathy in breast cancer patients.

## Background:

Systemic chemotherapy is an important therapeutic approach for breast cancer management where combinations of chemotherapeutic drugs including anthracyclines, platinum and taxanes treatment schedule have been widely adopted in the standard therapeutics [1, 2-3]. A different study showed that, chemotherapy drugs (DRG) are the most active class of cytotoxic agents for treatment of both early and advanced breast cancers [4]. Among chemotherapy-DRG, a combination of doxorubicin & paclitaxel is used as standard regimen against advanced breast cancer [4]. Studies have also concluded that, this chemotherapy-DRG can kill malignant cells. They can cause deleterious effects of normal healthy cells and cause adverse toxicity reactions too. Almost all chemotherapy agents can cause severe after effects (acute toxicity) in patients treated with chemotherapy where HEM and N-HEM adverse reactions are prominent [4, 5 -6]. Despite all the advances that have happened in the recent times, the outcome predictions of chemotherapy pattern cannot be generalized for all patients. Both the treatment responses and toxicity experienced are varied and unpredictable in each patient [7, 8, 9- 10]. Therefore, it is important to understand pharmacokinetic susceptibility of each individual towards the efficacy and toxicity of chemotherapy-DRG. The pharmacogenomics studies evidenced that functional gene polymorphisms encoding drug metabolizing enzymes (MT-E) can influence therapeutic (TPT) efficiency and treatment outcomes of different of chemotherapy-DRG which can lead to therapeutic failure and adverse toxicity effects [11, 12-13].

Studies have also shown that, there have been more than 2000 polymorphisms identified in cytochrome family genes which are reported to determine treatment response or toxicity as the variant genotypes of metabolizing enzymes encoding genes can alter activity of drug metabolizing enzymes which may lead to anomalous drug MT [11]. It has been also evident from earlier findings that the polymorphisms of majority of cytochrome genes are associated with therapeutic failure and chemotherapy induced severe toxicity reactions [11, 14-15]. Some of the earlier studies provided an association of CYP1A1*2A, CYP1A1*2C, CYP1B1*3 and CYP1B1*4-polymorphisms with platinum based chemotherapy response in lung cancer [16-17]. A study have showed that, the CYP1A1 (rs1048943) polymorphisms was significantly associated chemotherapy response towards platinum based chemotherapy in cervical cancer (CC) [18]. Furthermore, studies have shown that, polymorphisms of CYP1B1 may also contribute to the treatment response and survival of various CC patients [17, 19, 20-21]. A study on breast cancer showed that, there was an association of CYP1B1*3-polymorphisms with microsites reactions in response to paclitaxel based chemotherapy [22]. Similarly another studies showed association of gene polymorphisms of CYP1B1 with higher grade cardio toxicities in ovarian cancer (OC) patients [23, 24]. Studies also shown that, the CYP2C family genes including CYP2C8, CYP2C9, play an important role in MT of commonly used anticancer drugs [25, 26- 27]. Another study showed that, polymorphisms variants of CYP2C8*2, CYP2C8*3, CYP2C9*2, CYP2C9*3 genes showed there was a negative association with therapeutic response towards neo-adjuvant chemotherapy in breast cancer patients [8].

Other studies also showed that, there was a significant association of CYP2C9-polymorphisms in MT the therapeutic outcomes of chemotherapy-DRG in head and neck squamous cell carcinoma (SQ-CC) [28, 29]. Studies have also concluded that, polymorphisms of CYP2C8*3 significantly induce HEM-TC such as neutropenia in OC patients (P) in response to platinum and taxane (PL-TX) based chemotherapy [30, 31- 32]. The CYP2C9 (rs1057910) polymorphisms showed significant contribution in reduced response towards platinum and taxane based chemotherapy-DRG in OCP [33]. Conversely other studies showed non-significant impact of CYP1A1*2C, CYP1B1*4, CYP2D6*1A, CYP2E1*6, CYP2E1*7B polymorphisms with both platinum and taxane based chemotherapy response in in non-small cell lung carcinoma patients (CLC-P) [15, 17, 34]. Similarly, another study has shown that, there was no significant association of CYP1A1-polymorphisms was observed in response to chemotherapy-DRG-TC reactions & overall survival of acute lymphoblastic leukemia patients (LP-LK-P) [35]. Additionally, no association of CYP1A1 (rs4646903, rs1048943) polymorphisms was noted with PL based chemotherapy response in CC-P treated with cisplatin (CSP) [36]. The literature studies showed that, the polymorphisms of CYP1B1 showed no association with therapeutic response, outcomes and chemotherapy- toxicity in OC-P [23, 37]. Some other cohort studies showed non-significant correlation of CYP2C8 and CYP2C9 gene polymorphisms with paclitaxel plus CSP based chemotherapy outcomes and CPT induced toxicity in OC-P [23]. Therefore, it is of interest to assess the correlation of CYP1A1, CYP1B1, CYP2C genotypes with treatment efficacy and clinical outcomes in breast cancer patients administered with doxorubicin and paclitaxel based chemotherapy.

## Materials and Methods:

The current study included 200 patients in the Department of Oncology, KHMRC. A detailed clinic-pathological (CL-PATH) and demographic (DMOG) features along with follow up data of the patients were recorded. Of these patients, 104 patients were treated primarily with doxorubicin followed by paclitaxel and 96 patients were 1st treated with paclitaxel thereafter Doxorubicin. The C-TPT effects were determined after every chemotherapy cycle through blood testing (BD-TT). Patients were administered 4 cycles of combination chemotherapy with doxorubicin and Cyclophosphamide (CL-SP-AM), followed by 4 cycles of 3 weekly paclitaxel. After receiving 1st cycle of chemotherapy in each schedule, patient was followed again between 10th to 14th days after chemotherapy for assessing chemotherapy related toxicity. The patients administered chemotherapy and observed assessment of treatment response and acute toxicity evaluation. The chemotherapy induced HEM and N-HEM- toxicity were recorded and classified according to NCI-CTC Criteria. 5ml of whole blood from each patient was collected in sterile EDTA containing vacationer after receiving informed consent. Genomic DNA extraction was carried out from the peripheral blood sample using HipurA® Blood genomic DNA miniprep purification kit. (Cat no. MB504-250PR) (HI Media Laboratories) following the manufacturer's instructions. The genotyping of CYP450 enzyme genes including CYP1A1*2A, CYP1B1*3, CYP2C8*2, CYP2C8*3, CYP2C9*2, CYP2C9*3, were performed PCR restriction fragment length polymorphisms (PCR-RFLP). The PCR amplification were carried out separately in 20 micro liter (µL) reaction mixtures containing 1X PCR buffer 0.2 mM each dNTP, 10 picomole (pmol) of each primers (IDT technologies), 1U Taq DNA polymerase (GeNei, Merck Bioscience) and 100 nanogram (ng) of purified genomic DNA. The primer sequence used to amplify the CYP450 genes are shown in Table 1.

## Inclusion criteria:

[1] Histopathology confirm report

[2] Diagnosed with breast cancer and planned for standard chemotherapy (Doxorubicin and paclitaxel).

## Exclusion criteria:

[1] Patients with no pathological diagnosis

[2] Incomplete treatment

[3] Incomplete follow-up

[4] Patients with other comorbidities

[5] Abnormal liver

[6] Renal function tests

## Statistical analyses:

All tests were carried out using SPSS 11 Software. The relative risk, Odds Ratio (OR) and corresponding 95% confidence intervals (CI) were determined through unconditional multiple logistic regression (M-LR). The p values <0.05 were considered as statistically significant.

## Results:

Table 2 shows that, severe toxicity (grade <1) AM, 25 patients showed severe NP, 24 patients showed FB-NP & 7 patients faced TMCP. The severe N-HEM-TC with grade <1 were recorded as mucositis in 16 patients, CINV in 34 patients, fatigue in 37 patients, body ache in 15 patients and peripheral neuropathy in 5 patients after treatment with doxorubicin -chemotherapy. Table 3 shows that, rs1056836 SNP of CYP1B1 showed negative association with protective effects in BC-P in response to MCO reactions (OR=0.14, 95% CI: 0.03-0.54; p=0.004). The ORs with 95% CI of other SNPs for their correlation with MCO were: (CYP1A1 (rs1048943) (OR=2.00, 95% CI: 0.64-06.26; p=0.0229), CYP2C8*2(rs11572103) (OR=0.56, 940-3.68; p=0.723), CYP2C9*2 (rs1799853) (OR=0.31, 95% CI: 0.01-5.83; p=0.439), CYP2C9*3 (rs1057910) (OR=0.24, 95% CI: 0.03-1.95; p=0.182). We noted no association of gene polymorphisms of other CYP450 genes with CINV in BC-P. Table 4 shows that, significant negative association of variant (G/C) genotype of CYP1B1 (rs1056836) with H-PATH TNM grade>II (OR=0.56, 95% CI: 0.32-0.99; p=0.047) whereas other genotypes of CYP1A1 and CYP2C genotypes showed no association with H-PATH confirmed TNM grade >II. None of the genotype of CYP1A1, CYP1B1, CYP2C showed association with clinically confirmed TNM grade >II of the BC-P. The results also showed that CYP1B1 (G>C) polymorphisms showed significant negative association with ER/PR HR-S of breast cancer patients (OR=0.53, 95% CI: 0.29-0.94; p=0.031) whereas the genotype distribution of other genotypes showed no association with ER/PR or Her2 hormone receptors respectively.

## Discussion:

Several pharmacogenomics studies revealed that the patient's response towards different chemotherapy-DRG is not similar because of diverse genetic susceptibility of each individual towards the treatment response [11, 12-13]. The pharmacogenomics studies evidenced that polymorphisms of CYP450 genes encoding CYP450 enzymes could influence therapeutic efficiency and treatment outcomes of different chemotherapy-DRG [7, 8, 9- 10]. Studies have also shown that, the CYP1A1 gene polymorphisms was significantly studied for their association with C-TPT response towards platinum and taxane and based chemotherapy in different forms of cancer [16, 17-18].

When we studied the polymorphisms of CYP1A1 (rs1048943) in response to chemotherapy, we observed that CYP1A1 variant allele showed negative association with peripheral neuropathy in BC-P when treated with paclitaxel based chemotherapy (OR=0.35, 95% CI: 0.15-0.84; p=0.019). Similarly, CYP1B1*3 polymorphisms & its association with adryamycin and paclitaxel based chemotherapy noted negative association with protective effects of CYP1B1 (rs1056836) with MCO (OR=0.14, 95% CI: 0.03-0.54; p=0.004) in doxorubicin based chemotherapy and peripheral neuropathy in paclitaxel based chemotherapy in BC-P (OR=0.41, 95% CI: 0.17-0.96; p=0.040). These results are in contrast to the other studies reported a positive association of CYP1B1*3 polymorphisms with chemotherapy response and severe toxicity in breast cancer & OC [22, 23- 24].

In contrast to these findings, other researchers depicted significant association of CYP2C8*3 with HEM-TC toxicity such as neutropenia in ovarian cancer patients in response to platinum and taxane based chemotherapy [30, 31-32]. The CYP2C9 (rs1057910) polymorphism showed significant contribution in reduced response towards platinum based C-TPT-DRG in OC-P [33]. CYP2C9-polymorphisms modulate therapeutic outcomes of chemotherapy drugs in head and neck SQ-CC [28- 29]. Similarly, no significant association of CYP1A1-polymorphisms was observed in response to C-TPT- DRG-TC reactions and overall survival of acute LP-LK-P [35]. No association of CYP1A1 (rs4646903, rs1048943) polymorphisms was noted with PL based chemotherapy response in CC-P treated with cisplatin [36]. The literature studies showed that the polymorphisms of CYP1B1 showed no association with therapeutic response, outcomes and CPT chemotherapy toxicity in OC-P [23, 37].

Some other cohort studies showed non-significant correlation of CYP2C8 and CYP2C9 gene polymorphisms with paclitaxel plus cisplatin based chemotherapy outcomes & chemotherapy induced toxicity in OC-P [23]. There is a strong link between CYP2C9*2 and both hematological and non-hematological side effects of Adriamycin-based chemotherapy in a certain group of breast cancer patients. It was found that the CYP2C19*2 polymorphic variant genotype was strongly linked to anemia, neutropenia, and thrombocytopenia after Adriamycin treatment. The CYP17 polymorphism was strongly linked to body pain and peripheral neuropathy in people who were getting paclitaxel-based chemotherapy. So, they came to the conclusion that this is the first study of its kind to look at how chemotherapy based on Adriamycin affects metabolic gene polymorphisms in people with breast cancer [38].

While CYP2C19*2 rs4244285 possessed a significantly decreased risk (OR: 0.53, 95% CI: 0.33-0.85 P 0.009) of CC in the studied rural population, the CYP1B1*3 rs1056836 (Leu4326Val) polymorphism showed a significantly raised risk (OR = 3.28; 95% CI: 2.18-4.94; P 0.0001). A study found that the rs10244285 SNP of CYP2C19*2 lowers the risk of cancer in the group that was looked at, while the rs1056836 SNP of CYP1B1*3 raises the risk of cancer [39].

## Conclusion:

Data shows negative association of CYP1B1 with doxorubicin based chemotherapy induced N-HEM toxicity including mucositis and peripheral neuropathy in paclitaxel based chemotherapy in breast cancer patients of the selected population. Moreover, polymorphisms of CYP2C8 and CYP2C9 showed no association with either of hematological or N-HEM- toxicity in response to selected chemotherapy regime in breast cancer patients. Furthermore longitudinal studies are recommended to validate the results of our study.

## Figures and Tables

**Table 1:** The list of candidate ABCB genes selected

**Gene/ Genotype**	**RS number**	**Nucleotide change**	**Primer Sequence (Forward/Reverse)**	**PCR product**	**Digestion conditions**	**Dominant (Wild type)**	**Heterozygous**	**Recessive (Mutant)**
CYP1A1	rs1048943	(A>G)	FP: 5'- AAA GGC TGG GTC CAC CCT CT -3'	322 bp	1 Unit of NcoI	250 bp	322 bp	322 bp
Ex-7 A4889G			RP: 5'- AAA GAC CTC CCA GCG GGC CA-3'		Incubation at 37°C for 1h	72 bp	250 bp	72 bp
CYP1B1	rs1056836	(C>G)	FP: 5'-TTG GCC CTG AAA TCG CAC CGG T-3'	240 bp	1 Unit of BseNI	194 bp	240 bp	240 bp
Ex-3 C4326G			RP: 5'-CCA AGG ACA CTG TGG TTT TTG TCA AGC AG-3'		Incubation at 37°C for 1h	46 bp	194 bp	46 bp
CYP2C8*2	rs11572103	(T>A)	FP: 5'-AAA GTA AAA GAA CAC CAA GC-3'	167 bp	1 Unit of Kzo9I	69 bp	NIL	98 bp
Ex5 T805A			RP: 5'-AAA CAT CCT TAG TAA ATT ACA-3'		Incubation at 37°C for 1h	65 bp	69 bp	33 bp
CYP2C8*3	rs11572080	(G>A)	FP: 5'- AGG CAA TTC CCC AAT ATC TC-3'	467 bp	1 Unit of BseRI	310 bp	NIL	356 bp
Ex3 G416A			RP: 5'-CAG GAT GCG CAA TGA AGA C-3'		Incubation at 37°C for 1h	111 bp	111 bp	46 bp
CYP2C9*2	rs1799853	(C>T)	FP: 5'-CAC TGG CTG AAA GAG CTA ACA GAG-3'	372 bp	1 Unit of AspS9I	179 bp	NIL	253 bp
Ex-3 C430T			RP: 5'-GTG ATA TGG AGT AGG GTC ACC CAC-3'		Incubation at 37°C for 1h	119 bp	119 bp	74 bp
CYP2C9*3	rs1057910	(A>C)	FP:5'-AGG AAG AGA TTG AAC GTG TGA-3'	130 bp	1 Unit of ErhI	104 bp	NIL	130 bp
Ex-7 A1075C			RP: 5'GGC AGG CTG GTG GGG AGA AGG CCA A-3'		Incubation at 37°C for 1h	26 bp		

**Table 2:** Univariate analysis of candidate SNPS of cytochrome p450

		**Anemia(AM)**			
**Gene Name**	**Genotype**	**Grade ≤1**	**Grade >1**	**OR (95% CI)**	**p value**
SNP		(n=81)	(n=23)		
CYP1A1	A/A	38	9	1 (Reference)	
rs1048943	A/G+G/G	43	14	1.37 (0.53-3.53)	0.508
CYP1B1	C/C	44	13	1 (Reference)	
rs1056836	C/G+G/G	37	10	0.91 (0.35-2.32)	0.851
CYP2C8*2	T/T	77	23	1 (Reference)	
rs11572103	T/A+A/A	4	0	0.36 (0.01-7.05)	0.505
CYP2C8*3	G/G	55	14	1 (Reference)	
rs11572080	G/A+A+A	26	9	1.35 (0.52-3.54)	0.529
CYP2C9*2	C/C	74	23	1 (Reference)	
rs1799853	C/T+T/T	7	0	0.21 (0.01-3.84)	0.293
CYP2C9*3	A/A	66	18	1 (Reference)	
rs1057910	A/C+C/C	15	5	1.22 (0.39-3.81)	0.729
		**Neutropenia(NP)**			
		(n=79)	(n=25)		
CYP1A1	A/A	37	10	1 (Reference)	
rs1048943	A/G+G/G	42	15	1.32 (0.52-3.29)	0.55
CYP1B1	C/C	40	17	1 (Reference)	
rs1056836	C/G+G/G	39	8	0.48 (0.18-1.24)	0.132
CYP2C8*2	T/T	75	25	1 (Reference)	
rs11572103	T/A+A/A	4	0	0.32 (0.01-6.32)	0.461
CYP2C8*3	G/G	55	14	1 (Reference)	
rs11572080	G/A+A+A	24	11	1.80 (0.71-4.53)	0.212
CYP2C9*2	C/C	73	24	1 (Reference)	
rs1799853	C/T+T/T	6	1	0.50 (0.05-4.42)	0.538
CYP2C9*3	A/A	63	21	1 (Reference)	
rs1057910	A/C+C/C	16	4	0.75 (0.22-2.49)	0.639
		**Febrile Neutropenia(FB-NP)**			
		(n=80)	(n=24)		
CYP1A1	A/A	37	10	1 (Reference)	
rs1048943	A/G+G/G	43	14	1.20 (0.47-3.03)	0.692
CYP1B1	C/C	42	15	1 (Reference)	
rs1056836	C/G+G/G	38	9	0.66 (0.26-1.69)	0.389
CYP2C8*2	T/T	76	24	1 (Reference)	
rs11572103	T/A+A/A	4	0	0.34 (0.01-6.67)	0.482
CYP2C8*3	G/G	54	15	1 (Reference)	
rs11572080	G/A+A+A	26	9	1.24 (0.48-3.22)	0.649
CYP2C9*2	C/C	73	24	1 (Reference)	
rs1799853	C/T+T/T	7	0	0.20 (0.01-3.63)	0.276
CYP2C9*3	A/A	62	22	1 (Reference)	
rs1057910	A/C+C/C	18	2	0.31 (0.06-1.46)	0.139
		**Thrombocytopenia(TMCP)**			
		(n=97)	(n=7)		
CYP1A1	A/A	45	2	1 (reference)	
rs1048943	A/G+G/G	52	5	2.16 (0.40-11.69)	0.37
CYP1B1	C/C	53	4	1 (reference)	
rs1056836	C/G+G/G	44	3	0.90 (0.19-4.25)	0.897
CYP2C8*2	T/T	93	7	1 (reference)	
rs11572103	T/A+A/A	4	0	2.11 (0.09-47.48)	0.638
CYP2C8*3	G/G	66	3	1 (reference)	
rs11572080	G/A+A+A	31	4	2.83 (0.59-13.46)	0.188
CYP2C9*2	C/C	90	7	1 (reference)	
rs1799853	C/T+T/T	7	0	0.80 (0.04-15.49)	0.885
CYP2C9*3	A/A	78	6	1 (reference)	
rs1057910	A/C+C/C	19	1	0.68 (0.07-6.02)	0.732

**Table 3:** Risk of DXR-CTP induced severe TC of n-hem reactions in BC-p.

		**Mucositis (MCO)**			
**Gene Name**	**Genotype**	**Grade ≤1**	**Grade >1**	**OR (95% CI)**	**p value**
SNP		(n=88)	(n=16)		
CYP1A1	A/A	42	5	1 (Reference)	
rs1048943	A/G+G/G	46	11	2.00 (0.64-6.26)	0.229
CYP1B1	C/C	34	13	1 (Reference)	
rs1056836	C/G+G/G	54	3	0.14 (0.03-0.54)	0.004*
CYP2C8*2	T/T	84	16	1 (Reference)	
rs11572103	T/A+A/A	4	0	0.56 (0.02-11.08)	0.709
CYP2C8*3	G/G	59	10	1 (Reference)	
rs11572080	G/A+A+A	29	6	1.22 (0.40-3.68)	0.723
CYP2C9*2	C/C	78	16	1 (Reference)	
rs1799853	C/T+T/T	7	0	0.31 (0.01-5.83)	0.439
CYP2C9*3	A/A	69	15	1 (Reference)	
rs1057910	A/C+C/C	19	1	0.24 (0.03-1.95)	0.182
		**CINV**			
		(n=70)	(n=34)		
CYP1A1	A/A	27	20	1 (Reference)	
rs1048943	A/G+G/G	43	14	0.43 (0.19-1.01)	0.053
CYP1B1	C/C	38	19	1 (Reference)	
rs1056836	C/G+G/G	32	15	0.93 (0.41-2.13)	0.787
CYP2C8*2	T/T	66	34	1 (Reference)	
rs11572103	T/A+A/A	4	0	0.21 (0.01-4.09)	0.306
CYP2C8*3	G/G	47	22	1 (Reference)	
rs11572080	G/A+A+A	23	12	1.11 (0.47-2.64)	0.805
CYP2C9*2	C/C	64	33	1 (Reference)	
rs1799853	C/T+T/T	6	1	0.32 (0.03-2.79)	0.305
CYP2C9*3	A/A	68	30	1 (Reference)	
rs1057910	A/C+C/C	16	4	0.56 (0.17-1.83)	0.344
		**Fatigue(FTG)**			
		(n=67)	(n=37)		
CYP1A1	A/A	31	16	1 (Reference)	
rs1048943	A/G+G/G	36	21	1.132 (0.50-2.53)	0.766
CYP1B1	C/C	35	22	1 (Reference)	
rs1056836	C/G+G/G	32	15	0.74 (0.33-1.68)	0.479
CYP2C8*2	T/T	64	36	1 (Reference)	
rs11572103	T/A+A/A	3	1	0.59 (0.05-5.90)	0.655
CYP2C8*3	G/G	45	24	1 (Reference)	
rs11572080	G/A+A+A	22	13	1.10 (0.47-2.58)	0.812
CYP2C9*2	C/C	60	37	1 (Reference)	
rs1799853	C/T+T/T	7	0	0.10 (0.006-1.93)	0.13
CYP2C9*3	A/A	56	28	1 (Reference)	
rs1057910	A/C+C/C	11	9	1.63 (0.60-4.40)	0.33
		**Body ache(BAC)**			
		(n=89)	(n=15)		
CYP1A1	A/A	39	8	1 (Reference)	
rs1048943	A/G+G/G	50	7	0.68 (0.22-2.04)	0.495
CYP1B1	C/C	46	11	1 (Reference)	
rs1056836	C/G+G/G	43	4	0.38 (0.11-1.31)	0.128
CYP2C8*2	T/T	86	14	1 (Reference)	
rs11572103	T/A+A/A	3	1	2.04 (0.19-21.10)	0.547
CYP2C8*3	G/G	60	9	1 (Reference)	
rs11572080	G/A+A+A	29	6	1.37 (0.44-4.24)	0.575
CYP2C9*2	C/C	82	15	1 (Reference)	
rs1799853	C/T+T/T	7	0	0.35 (0.01-6.53)	0.485
CYP2C9*3	A/A	71	13	1 (Reference)	
rs1057910	A/C+C/C	18	2	0.60 (0.12-2.93)	0.534
		**Peripheral Neuropathy(PP-NP)**			
		(n=99)	(n=5)		
CYP1A1	A/A	45	2	1 (Reference)	
rs1048943	A/G+G/G	54	3	1.25 (0.20-7.81)	0.811
CYP1B1	C/C	54	3	1 (Reference)	
rs1056836	C/G+G/G	45	2	0.80 (0.12-4.99)	0.811
CYP2C8*2	T/T	95	5	1 (Reference)	
rs11572103	T/A+A/A	4	0	1.92 (0.09-40.55)	0.672
CYP2C8*3	G/G	68	1	1 (Reference)	
rs11572080	G/A+A+A	31	4	8.77 (0.94-81.77)	0.056
CYP2C9*2	C/C	92	5	1 (Reference)	
rs1799853	C/T+T/T	7	0	1.12 (0.05-22.27)	0.94
CYP2C9*3	A/A	79	5	1 (Reference)	
rs1057910	A/C+C/C	20	0	0.35 (0.01-6.63)	0.486

**Table 4:** Association of CYP1A1, CYP1B1and CYP2C gene polymorphisms with demographic and clinic-pathological factors of BC patients

**Characteristics**	**CYP1A1 (rs1048943)**		**CYP1B1 (rs1056836)**		CYP2C8*2 (rs11572103)	
	**A/A**	**A/G+A/A**	**G/G**	**G/C+C/C**	**T/T**	**T/A+A/A**
	**No (%)**	**No (%)**	**No (%)**	**No (%)**	**No (%)**	**No (%)**
Age						
≤ 40	15 (7.50)	28 (14.00)	21 (10.50)	22 (11.00)	42 (21.00)	1 (0.50)
>40	81 (40.50)	76 (38.00)	79 (39.50)	78 (39.00)	148 (74.00)	9 (4.50)
OR (95% CI)	1 (Reference)	0.50 (0.24-1.01)	1 (Reference)	0.94 (0.47-1.85)	1 (Reference)	2.55 (0.31-20.73)
p value		0.054		0.863		0.38
**BMI Kg/m^2^**						
≤ 25	65 (32.50)	57 (28.50)	64 (32.00)	58 (29.00)	113 (56.50)	9 (4.50)
>25	31 (15.50)	47 (23.50)	36 (18.00)	42 (21.00)	77 (38.50)	1 (0.50)
OR (95% CI)	1 (Reference)	1.72 (0.97-3.07)	1 (Reference)	1.28 (0.72-2.27)	1 (Reference)	0.16 (0.02-1.31)
p value		0.062		0.384		0.088
**Clinical TNM Grade**						
≤ Stage II	52 (26.00)	50 (25.00)	55 (27.50)	47 (23.50)	101 (50.50)	1 (0.50)
> Stage II	44 (22.00)	54 (27.00)	45 (22.50)	53 (26.50)	89 (44.50)	9 (4.50)
OR (95% CI)	1 (Reference)	1.27 (0.73-2.22)	1 (Reference)	1.37 (0.79-2.40)	1 (Reference)	10.21 (1.26-82.21)
p value		0.389		0.258		0.029
**Histopathological(H-PATH) TNM Grade**						
≤ Stage II	39 (19.50)	51 (25.50)	38 (19.00)	52 (26.00)	89 (44.50)	1 (0.50)
> Stage II	57 (28.50)	53 (26.50)	62 (31.00)	48 (24.00)	101 (50.50)	9 (4.50)
OR (95% CI)	1 (Reference)	0.71 (0.40-1.24)	1 (Reference)	0.56 (0.32-0.99)	1 (Reference)	7.93 (0.98-63.83)
p value		0.232		0.047*		0.051
**Hormone Receptor Status(HR-S)**						
ER/PR +ve	41 (20.50)	42 (21.00)	33 (16.50)	50 (25.00)	80 (40.00)	3 (1.50)
ER/PR -ve	55 (27.50)	62 (31.00)	67 (33.50)	50 (25.00)	110 (55.00)	7 (3.50)
OR (95% CI)	1 (Reference)	1.10 (0.62-1.93)	1 (Reference)	0.53 (0.29-0.94)	1 (Reference)	1.69 (0.42-6.76)
p value		0.739		0.031*		0.453
Her2 +ve	20 (10.00)	12 (6.00)	17 (8.50)	15 (7.50)	31 (15.50)	1 (0.50)
Her2 -ve	76 (38.00)	92 (46.00)	83 (41.50)	85 (42.50)	159 79.50)	9 (4.50)
OR (95% CI)	1 (Reference)	2.01 (0.92-4.39)	1(Reference)	1.16 (0.54-2.47)	1 (Reference)	1.75 (0.21-14.35)
p value		0.076		0.699		0.6
